# Using sentinel surveillance system data to characterize severe malaria illness and quality of malaria case management among hospitalized patients in Kenya, 2017–2024

**DOI:** 10.1186/s12936-025-05738-3

**Published:** 2026-01-14

**Authors:** Megumi Itoh, Naomi Lucchi, Jonathan Schultz, George O. Agogo, Peninah Munyua, Duncan Chege, Doris Naitore Mwenda, Steve Akoth, Victor Sumbi, Mildred Shieshia, Regina Kandie, Edwin Oluoch Onyango, Jonas Z. Hines

**Affiliations:** 1 Malaria Branch, Division of Parasitic Diseases and Malaria, National Center for Emerging and Zoonotic Diseases, Centers for Disease Control and Prevention, Nairobi, Kenya; 2 President’s Malaria Initiative, United States Agency for International Development, Nairobi, Kenya; 3 Division of Global Health Protection, Global Health Center, Centers for Disease Control and Prevention, Nairobi, Kenya; 4 Malaria Branch, Division of Parasitic Diseases and Malaria, National Center for Emerging and Zoonotic Diseases, Centers for Disease Control and Prevention, Kisumu, Kenya; 5 ICAP at Columbia University, Nairobi, Kenya; 6 National Malaria Control Programme, Kenya Ministry of Health, Nairobi, Kenya

**Keywords:** Malaria, Sentinel surveillance, Quality of health care, Kenya, Africa

## Abstract

**Background:**

In Kenya, limited clinical data on hospitalized malaria patients restricts insights into disease severity and care quality. Using data from the Integrated Facility-based Surveillance (IFBS) system—a sentinel surveillance platform for febrile illnesses across twelve facilities—the assessment focused on risk factors for severe illness and mortality, diagnostic accuracy of microscopy, and adherence to severe malaria treatment guidelines.

**Methods:**

Analysis of IFBS data obtained from June 2017 to July 2024 was performed using bivariable logistic regression to identify factors linked to severe illness and deaths. Microscopy results were compared with PCR results to assess diagnostic concordance. Evaluation also included whether patients received parasitological confirmation before treatment and if severe cases received IV artesunate followed by artemether-lumefantrine (AL), per standard guidelines.

**Results:**

Among 8,487 inpatients, 2,197 (25.9%) tested positive for malaria by either microscopy or rapid diagnostic test; among malaria cases, 713 (32.5%) had severe disease and 16 (0.7%) died. Infants had greater odds of severe illness compared to older ages (odds ratio [OR] was < 1.0 for other age groups compared to ≤ 1 year-old). Both severe illness and death were associated with fever duration of ≥ 5 days compared to ≤ 1 day (ORs: 3.67 and 8.00, respectively) and having been referred from another facility (ORs: 3.01 and 3.15, respectively). Positive microscopy at the health facility was PCR negative in 21% of patients. Only 15% of severe cases were documented to have received both IV artesunate and AL, while 17% received IV quinine.

**Conclusions:**

Modifiable factors that suggested delayed care-seeking were associated with worse malaria outcomes in Kenya. Furthermore, gaps in diagnostic accuracy and adherence to treatment protocols for severe malaria were observed during chart review. These findings point to the importance of behaviour change strategies as well as messaging in the community that promote timely care-seeking, referrals and follow-up, especially for the youngest children. Potential malaria over-diagnosis underscores the need for strengthening quality assured microscopy programs with adequate training of microscopists and properly functioning microscopes and reagents, as well as an external quality assurance programme that routinely provide feedback on performance and identify areas for improvement.

**Supplementary Information:**

The online version contains supplementary material available at 10.1186/s12936-025-05738-3.

## Background

Malaria control investments have significantly reduced disease burden in Kenya, with prevalence among children under give dropping from 11% in 2010 [1] to 6% in 2020 [2]. While severe malaria cases and mortality have generally declined, they have plateaued in recent years [3–6].Advancements in malaria surveillance have improved the quality of routine outpatient malaria data [7], but information on hospitalized patients remains sparse and incomplete across Kenya and more broadly in Africa [8, 9]. Routine data focus predominantly on uncomplicated malaria, resulting in limited availability of clinical presentation, treatment and outcome data for admitted patient with severe malaria [10].

The case fatality rate (CFR) is the only routinely reported indicator for malaria-related hospitalizations in Kenya, calculated by dividing facility malaria-related deaths by total malaria admissions [11]. However, this indicator has significant limitations due to incomplete data capture of admissions and discharge diagnoses in the inpatient setting. Consequently, the routinely collected surveillance data cannot provide the detailed clinical picture, including risk factors for severe presentation and mortality, typically obtained only through resource-intensive research studies or enhanced surveillance activities conducted at a limited number of health facilities.

The Integrated Facility-based Surveillance (IFBS) platform is a sentinel disease surveillance system spanning 12 health facilities across various malaria epidemiologic zones in Kenya (out of ~ 1600 public inpatient facilities) [12]. IFBS systematically enrolls patients with acute febrile illness (AFI), defined as an axillary temperature of ≥ 38.0 °C for ≤ 14 days, and collects clinical, laboratory and pathogen data, including malaria diagnosis confirmed by malaria rapid diagnostic testing (RDT), expert microscopy, and in some cases, by TaqMan™ Array Cards multiplex PCR testing (TAC). This unique, rich, multi-site dataset describes the clinical characteristics, treatments and outcomes of inpatients with malaria and co-morbidities. Specifically, IFBS allows for the identification of co-infections with non-*Plasmodium* pathogens, such as non-typhoidal *Salmonella* (NTS), which are known to be a significant contributor to severe disease and mortality, particularly among children with malaria [13–15].

Microscopy is the gold standard clinical diagnostic method for malaria in Kenya given its relative cost and implementation feasibility at the point-of-service in higher-level facilities. Malaria RDTs are used when microscopy is not possible, typically in lower-level facilities without laboratory infrastructure or adequately trained staff. Although facility laboratory staff receive pre-service training in malaria diagnostics, the reliability of routine microscopy is often constrained by resource limitations in external quality assurance (EQA) programmes [16].Because the IFBS platform conducts its own gold-standard malaria testing on all enrolled patients, it provides a unique opportunity to assess diagnostic accuracy by comparing routine clinical smears read by laboratory staff at the facilities (clinical smears) with those read by expert surveillance microscopists (surveillance smears). Furthermore, the IFBS data on treatments provided (antimalarials and empiric antibiotics) allow for the direct evaluation of adherence to clinical guidelines in the inpatient setting [17].

The study utilized IFBS data to pursue two primary objectives: (1) to assess factors associated with severe illness and mortality among patients with laboratory-confirmed malaria; and (2) to evaluate the quality of inpatient malaria case management by examining diagnostic accuracy and adherence to treatment guidelines.

## Methods

### Study design and sites

**Fig. 1:**
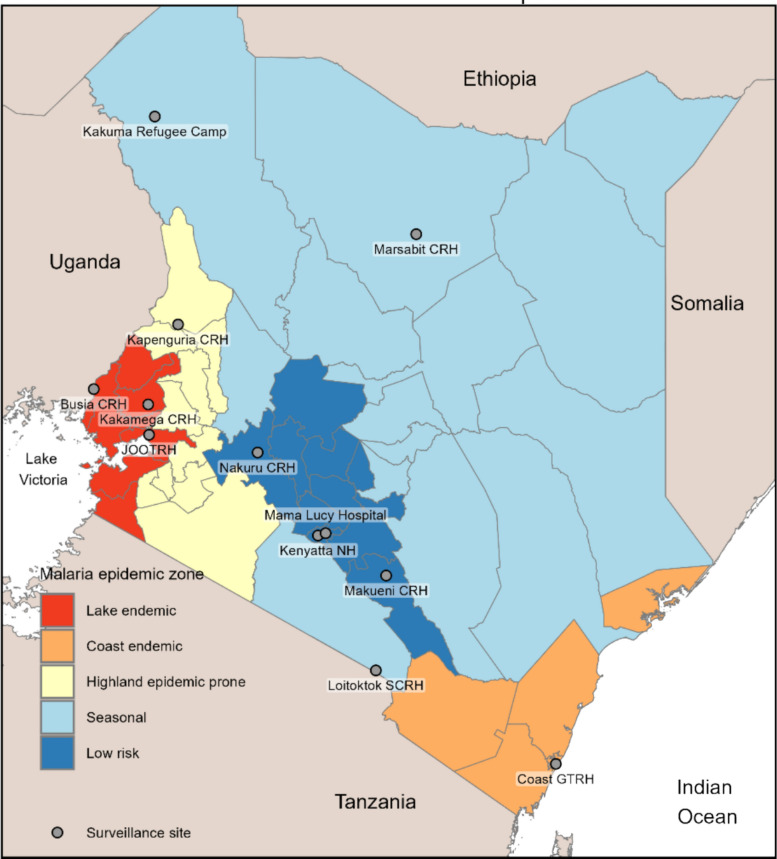
Location of surveillance sites and malaria epidemic zones in Kenya

Analysis utilized IFBS data from June 2017 to July 2024. IFBS initially monitored AFI at four facilities in 2017 and has since expanded its scope (e.g., mortality, antimicrobial resistance) and number of sites. This analysis concentrated on the clinical characteristics and hospitalization course of AFI patients diagnosed with malaria across 12 health facilities in Kenya during the study period (Fig. 1), acknowledging that the specific sites contributing data were not constant (Supplemental Table 1).

### Patient enrollment

IFBS procedures have been previously described [12]. Briefly, trained surveillance officers screened daily hospital admission logs (paediatric and adult medical wards) for the previous 24 h. Patients were eligible if they had an AFI, defined as a measured temperature ≥ 38.0 °C for ≤ 14 days based on medical record information. Although some sites also recruited patients ≥ 13 years old from outpatient clinics, we limited our analysis exclusively to inpatients given project’s objectives.

### Data collection and laboratory procedures

Following enrollment, surveillance officers interviewed the patient and extracted detailed medical history, clinical presentation, vital signs, physical exam, clinical management, routine lab results (including RDT/microscopy), and outcomes from their medical records, recording this information digitally. Regardless of clinical testing, 5 cc (2.5 cc for children under five) of venous blood was collected in ethylenediaminetetraacetic acid (EDTA) anticoagulant tubes. Onsite laboratory staff performed a surveillance RDT (Abbott Bioline™ Malaria Ag P.f/Pan) and prepared Giemsa-stained thin and thick blood smears following WHO procedures [18].Slides were sent weekly to Jaramogi Oginga Odinga Teaching & Referral Hospital in Kisumu, where certified expert microscopists analysed them. A subset of patients meeting criteria for undifferentiated fever (defined as AFI without evidence of lower respiratory tract infection, diarrhea, or another source of fever based on history and physical examination (e.g., meningitis, skin/soft tissue infection) underwent further testing: whole blood samples were stored at − 20 °C for up to seven days and transported to the CDC-supported laboratory at the Kenya Medical Research Institute (KEMRI) in Nairobi for multiplex PCR testing using TAC (ThermoFisher) (Supplemental Table 2). Note that TAC PCR was intended for surveillance only and was discontinued for IFBS RDT-positive patients after June 2023 as a resource savings measure.

### Definitions

To align with the study’s dual objectives, two distinct definitions for a positive malaria diagnosis were used. For the analysis of severe disease and mortality factors, a positive case was defined by a positive IFBS surveillance RDT or microscopy. Cases positive by TAC PCR test only without a positive parasitological result were considered submicroscopic and excluded, in line with national guidelines for clinical diagnosis [19]. For the analysis assessing adherence to MoH clinical guidelines, a positive case was defined by a positive routine clinical RDT or microscopy, as this was the result used for patient management.

While all inpatients technically meet the definition of “severe malaria” by virtue of being assessed by a clinician to require hospitalization, inclusion in the severe illness analysis required documentation of at least one of the following characteristics: physical exam findings of severe respiratory disease (i.e., oxygen saturation of ≤ 90% or supplemental oxygen requirement, stridor, nasal flaring, lower chest indrawing, or grunting), severe neurological disease (i.e., level of consciousness below “alert” on the Alert/Verbal/Pain/Unresponsive [AVPU] scale, convulsions, photophobia, nuchal rigidity, and bulging fontanelle), jaundice, petechial rash, or severe anaemia (haemoglobin < 7 g/dL) [20].Patient outcomes were dichotomized as discharged in stable condition or died; those with ambiguous outcomes (e.g., discharged against medical advice, transferred) were excluded from the mortality analysis.

### Statistical analysis

Demographic and baseline characteristics were described using frequencies and percentages, with differences tested via chi-square tests analysis. Bivariate logistic regression analyses were performed in R (v4.4.2) to ascertain factors associated with severe illness and mortality, assessing patient characteristics including sex, age, malaria epidemic zone, number of days of fever prior to presentation, prior care-seeking (including referrals from other health facilities and antimalarials taken prior to admission), pregnancy, HIV status, and malnutrition. Pregnancy status was self-reported for women aged 15–49 years, and HIV status was ascertained by self-report or the medical chart but was not confirmed by HIV testing. Malnutrition status was determined by age-based mid-upper arm circumference (MUAC) cut-offs for children aged < 5 years.

### Co-infection analysis

Malaria co-infection was defined as a *Plasmodium* species infection plus another non-*Plasmodium* pathogen detected by TAC PCR. The odds ratios were calculated for severe illness or mortality, comparing malaria mono-infection, co-infection, and other pathogens. This analysis included all patients who received TAC PCR, regardless of their malaria test result.

### Quality of care assessment

To evaluate the diagnostic accuracy of clinical and surveillance smears/RDTs, positive and negative predictive values (PPV/NPV) were calculated using the more sensitive PCR as the reference standard for detecting misdiagnosis (false positives). Treatment adherence was assessed by determining if patients with a clinical malaria diagnosis received antimalarials per national guidelines: injectable artesunate followed by artemether-lumefantrine (AL) for severe disease, AL alone for uncomplicated disease, or IV quinine if artesunate was unavailable [19]. The assessment also included two other key measures: (1) the proportion of patients receiving any antimalarial treatment without a parasitological diagnosis, and (2) the proportion of severe malaria cases receiving empiric antibiotics per WHO guidelines [21], particularly in moderate to high transmission areas, which is applicable in Kenya’s malaria endemic zones in western Kenya as well as on the coast.

### Ethics and project funding

The data were collected under a protocol approved by Kenya Medical Research Institute institutional review board. The project was reviewed by CDC, deemed not research, and was conducted consistent with applicable U.S. federal law and CDC policy (See e.g., 45 C.F.R. part 46.102(l)(2), 21 C.F.R. part 56; 42 U.S.C. §241(d); 5 U.S.C. §552a; 44 U.S.C. §3501 et seq.). The project was financially supported by the U.S. Centers for Disease Control and Prevention under the terms of cooperative agreement with ICAP at Columbia University (CoAg number: NU2HGH000033).

## Results

**Fig. 2:**
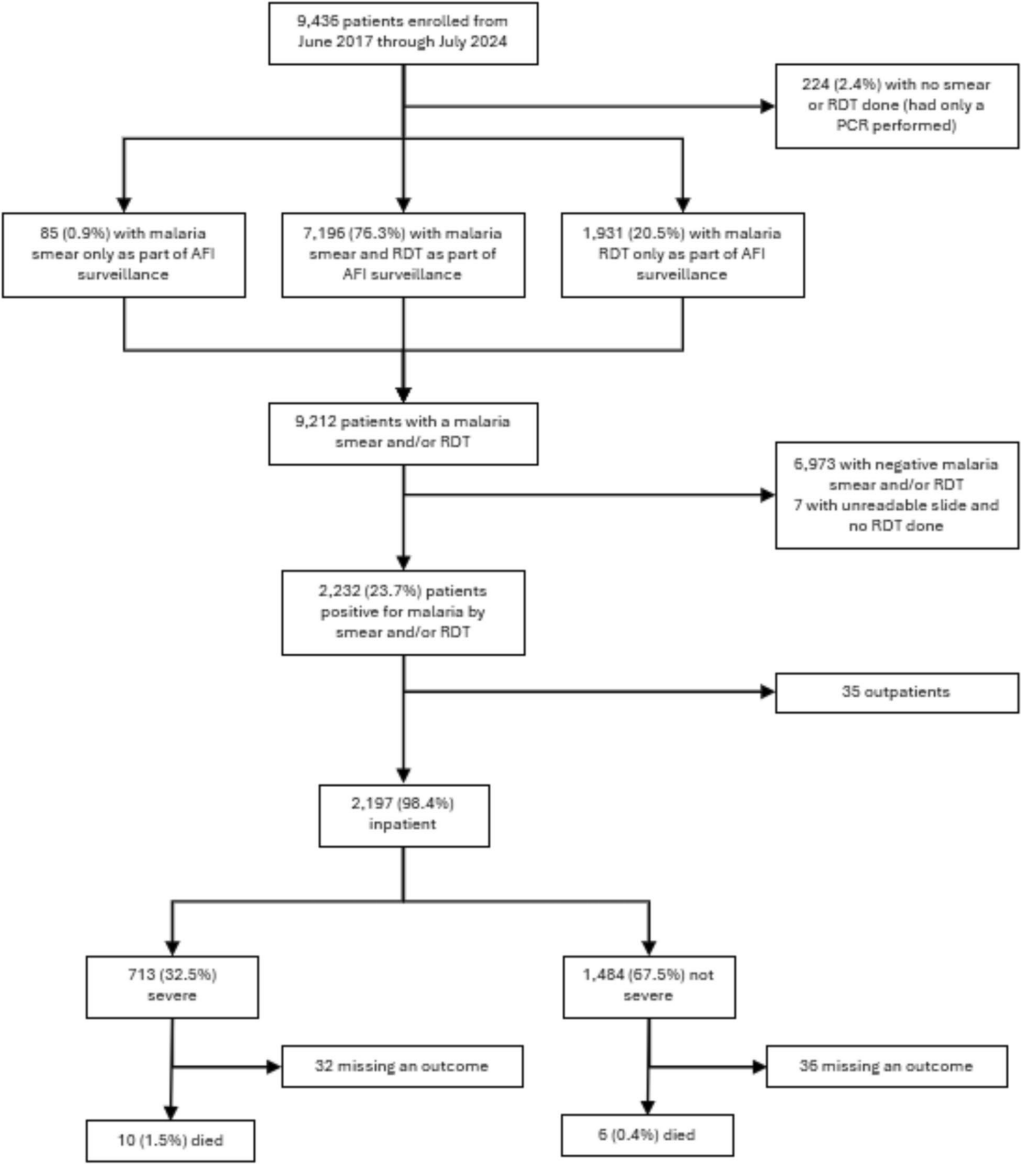
Flow chart of patients enrolled, malaria tests done by surveillance team and results, as well as clinical outcomes

In total, 9,436 patient records were available from the IFBS platform from June 2017 to July 2024 (Fig. 2). Of the 9,212 patients who received a surveillance smear or RDT, 2,232 (23.7%) tested positive for malaria. Among the 2,197 hospitalized patients with confirmed malaria, 713 (32.5%) met criteria for severe illness presentation and 16 (0.7%) resulted in death. The proportion of severe malaria was significantly higher in younger patients compared to older groups as follows: < 1 year (38.8%), 1–4 years (34.4%), 5–19 years (33.0%), ≥ 20 years (12.9%) (see Supplemental Table 3 for component severity variables).

### Factors associated with severe illness and mortality

The bivariate analysis (Table 1) revealed several factors associated with greater odds of severe illness presentation: very young age (< 1 year), longer fever duration before presentation, and having sought prior care (including facility referral and self-reported antimalarial use within the last 7 days). Similarly, increased odds of mortality were associated with longer fever duration, facility referral and prior antimalarial use. Finally, severe illness presentation itself was also associated with increased mortality among patients with malaria.

**Table 1:** Factors associated with severe illness and mortality among inpatients with AFI and positive malaria test

Characteristic		Severe illness presentation*			Mortality^†^		
	Overall, n (%) N = 2,197	Not severe, n (%) N = 1,484	Severe, n (%) N = 713	OR (95% CI)	Discharged, n (%) N = 2,113	Dead, n (%) N = 16	OR (95% CI)
Sex							
Female	984 (45%)	663 (45%)	321 (45%)	Ref	946 (45%)	9 (56%)	Ref
Male	1,213 (55%)	821 (55%)	392 (55%)	0.99 (0.82–1.18)	1,167 (55%)	7 (44%)	0.63 (0.22–1.70)
Age group (years)							
**≤ 1**	188 (9%)	115 (8%)	73 (10%)	Ref	185 (9%)	0 (0%)	Ref
1–4	1,134 (52%)	744 (50%)	390 (55%)	0.83 (0.60–1.14)	1,098 (52%)	5 (31%)	NC
5–19	681 (31%)	456 (31%)	225 (32%)	0.78 (0.56–1.09)	643 (30%)	7 (44%)	NC
≥ 20	194 (9%)	169 (11%)	25 (4%)	0.23 (0.14–0.38)	187 (9%)	4 (25%)	NC
Epi zone							
Lake endemic	941 (43%)	521 (35%)	420 (59%)	Ref	876 (41%)	9 (56%)	Ref
Coast endemic	200 (9%)	112 (8%)	88 (12%)	0.97 (0.72–1.32)	193 (9%)	4 (25%)	2.02 (0.54–6.26)
Highland epidemic prone	109 (5%)	62 (4%)	47 (7%)	0.94 (0.63–1.40)	108 (5%)	0 (0%)	NC
Seasonal	764 (35%)	687 (46%)	77 (11%)	0.14 (0.11–0.18)	756 (36%)	3 (19%)	0.39 (0.09–1.30)
Low risk	183 (8%)	102 (7%)	81 (11%)	0.99 (0.71–1.35)	180 (9%)	0 (0%)	NC
Days of fever (n miss = 21)							
≤ 1	425 (20%)	331 (23%)	94 (13%)	Ref	416 (20%)	1 (6%)	Ref
2–4	1,420 (65%)	976 (66%)	444 (63%)	1.60 (1.25–2.08)	1,364 (65%)	9 (56%)	2.74 (0.51–50.70)
≥ 5	331 (15%)	162 (11%)	169 (24%)	3.67 (2.69–5.05)	312 (15%)	6 (38%)	8.00 (1.36–151.00)
Pregnant‡ (n miss = 4)							
No	145 (82%)	127 (81%)	18 (90%)	Ref	141 (82%)	3 (100%)	Ref
Yes	32 (18%)	30 (19%)	2 (10%)	0.47 (0.07–1.75)	32 (18%)	0 (0%)	NC
HIV status¶ (n miss = 707)							
Negative	1,465 (98%)	961 (99%)	504 (98%)	Ref	1,410 (98%)	9 (90%)	Ref
Positive	25 (2%)	14 (1%)	11 (2%)	1.50 (0.66–3.32)	24 (2%)	1 (10%)	6.53 (0.35–36.80)
Malnourished﻿** (n miss = 196)							
Not malnourished	1,056 (94%)	686 (94%)	370 (93%)	Ref	1,023 (94%)	4 (100%)	Ref
Moderate	56 (5%)	31 (4%)	25 (6%)	1.50 (0.86–2.57)	56 (5%)	0 (0%)	NC
Severe	14 (1%)	12 (2%)	2 (1%)	0.31 (0.05–1.14)	14 (1%)	0 (0%)	NC
Received care before presentation (n miss = 21)							
No	1,444 (66%)	1,101 (75%)	343 (49%)	Ref	1,408 (67%)	9 (56%)	Ref
Yes	732 (34%)	371 (25%)	361 (51%)	3.12 (2.59–3.77)	684 (33%)	7 (44%)	1.60 (0.57–4.31)
Referred from another HF (n miss = 136)							
No	1,727 (84%)	1,236 (89%)	491 (73%)	Ref	1,696 (84%)	10 (63%)	Ref
Yes	334 (16%)	152 (11%)	182 (27%)	3.01 (2.37–3.83)	323 (16%)	6 (38%)	3.15 (1.07–8.54)
Took antimalarials ≤ 7 days (n miss = 32)							
No	1,768 (82%)	1,264 (86%)	504 (72%)	Ref	1,715 (82%)	8 (50%)	Ref
Yes	397 (18%)	203 (14%)	194 (28%)	2.40 (1.92–2.99)	368 (18%)	8 (50%)	4.66 (1.70–12.70)
Had a clinical malaria test﻿†† (n miss = 1)							
No	183 (8%)	111 (7%)	72 (10%)	Ref	177 (8%)	2 (13%)	Ref
Yes	2,013 (92%)	1,372 (93%)	641 (90%)	0.72 (0.53–0.99)	1,935 (92%)	14 (88%)	0.64 (0.18–4.10)
Disease severity							
Not severe					1,442 (68%)	6 (38%)	Ref
Severe					671 (32%)	10 (63%)	3.58 (1.32–10.60)

*Severe illness was defined as physical exam findings of severe respiratory process (i.e. oxygen saturation of ≤ 90% or supplemental oxygen requirement, stridor, nasal flaring, lower chest indrawing, or grunting), severe neurological process (i.e. level of consciousness below “alert” on the Alert/Verbal/Pain/Unresponsive [AVPU] scale, convulsions, photophobia, nuchal rigidity, and bulging fontanelle), jaundice, petechial rash, or severe anaemia defined as haemoglobin < 7 g/dL

^†^Outcome was only assessed among inpatients (N = 2,197), of which 68 were missing a known outcome

^‡^Pregnancy status self-reported from females aged 15–49 years (N = 181)

^¶^HIV status was self-reported or recorded in the medical chart. Testing status was not verified with an HIV test

**MUAC measured in children aged under 5 years (N = 1,332). In children 6 months to 4 years old, moderate malnutrition defined as MUAC 11.5–12.4 cm and severe malnutrition defined as MUAC < 11.5 cm. In children younger than 6 months, moderate malnutrition defined as < 11.0 cm

^††^Received either a RDT or blood smear as part of their routine clinical care at the health facility

CI: confidence interval, HF: health facility, NA: not applicable, NC: not calculated, OR: odds ratio, RDT: rapid diagnostic test, Ref: referent level

### Malaria co-infections and association with disease severity and mortality

TAC PCR was performed on 6,069 patients, detecting *P. falciparum* in 2,093 cases (34.5%) (Table 2). Limiting the cohort to inpatients with a positive surveillance RDT and/or positive blood smear (n = 129), *Plasmodium* spp. was detected in 94.3% (1219) of cases. The most common co-infections detected by TAC PCR were non-typhoid Salmonella (NTS) (2.0%), followed by HIV-1 (1.2%), *Rickettsia* (0.9%), and Dengue virus (0.9%). While there was no clear association between TAC PCR results and disease severity, the odds of mortality were higher for patients with malaria co-infection, detection of a non-*Plasmodium* pathogen, or a negative TAC result. Only the detection of a non-*Plasmodium* pathogen was statistically significant, though a negative TAC result showed near significance (Table 3).

**Table 2:** Pathogens detected on TaqMan Array Card of persons with acute febrile illness in Kenya, 2017–2024

Pathogen^*,^^†^	Inpatients with an RDT and/or smear positive for malaria (N = 1293)	All persons with AFI with TAC done (N = 6069)^‡^
*Plasmodium* spp*.*	1219 (94.3%)	2093 (34.5%)
HIV-1	16 (1.2%)	136 (2.2%)
Non-typhoid *Salmonella*	26 (2.0%)	73 (1.2%)
*Rickettsia*	12 (0.9%)	72 (1.2%)
Dengue	11 (0.9%)	52 (0.9%)
*Streptococcus pneumoniae*	5 (0.4%)	44 (0.7%)
*Brucella*	6 (0.5%)	42 (0.7%)
*Salmonella Typhi*	7 (0.5%)	38 (0.6%)
Leishmania	1 (0.1%)	34 (0.6%)
Chikungunya	4 (0.3%)	28 (0.5%)
*Bartonella*	1 (0.1%)	22 (0.4%)
*Leptospira*	2 (0.2%)	14 (0.2%)
*Coxiella burnetiid*	4 (0.3%)	10 (0.2%)
*Plasmodium vivax*	0 (0.0%)	5 (0.1%)
Rift Valley Fever virus	1 (0.1%)	3 (0.0%)
*Burkholderia pseudomallei*	0 (0.0%)	2 (0.0%)
Negative TAC	66 (5.1%)	3630 (59.8%)

*Results for targets for *Plasmodium falciparum* and *Plasmodium vivax* were not shown to avoid double-counting and because these targets were not added until November 2020. All *P. falciparum* and *P. vivax* detections were positive for the *Plasmodium* spp. target but not vice versa

^†^TAC assay was updated in November 2020 to add the following targets: *Burkholderia pseudomallei*, *Orientia tsutsugamushi*, Oropouche virus, *Plasmodium falciparum*, *Plasmodium vivax*, *Streptococcus pneumoniae*, and *Salmonella* paratyphi A

^‡^Includes all patients (inpatient and outpatient) who received a TAC test as part of AFI surveillance in Kenya

AFI: acute febrile illness; RDT: rapid diagnostic test; TAC: TaqMan Array Card

Of note, the following pathogens have not been detected on this platform in Kenya: Crimean-Congo hemorrhagic fever virus, Ebola virus (Zaire, Budibugyo, Sudan), Hepatitis E virus, Lassa virus, Marburg virus, Nipah virus, O'nyong-nyong virus, *Orientia tsutsugamushi,* Oropouche virus, *Salmonella paratyphi A, Trypanosomiasis brucei,* West Nile virus, Yellow Fever virus, *Yersinia pestis,* Zika virus

**Table 3:** Risk of severe disease and mortality based on TAC results (N = 6069)*

Category	Severe illness presentation				Mortality			
	Not severe	Severe	OR	95% CI	Discharged	Died	OR	95% CI
Malaria only	1346 (32%)	572 (30%)	Ref		1733 (35%)	23 (23%)	Ref	
Malaria coinfection	130 (3%)	45 (2%)	0.81	0.57, 1.15	150 (3%)	4 (4%)	2.01	0.58, 5.31
Other pathogen(s)	237 (6%)	109 (6%)	1.08	0.84, 1.38	**263 (5%)**	**15 (15%)**	**4.30**	**2.17, 8.27**
Negative	2458 (59%)	1,172 (62%)	1.12	1.00, 1.27	**2831 (57%)**	**59 (58%)**	**1.57**	**0.98, 2.60**

*This analysis is among all persons with a TAC result regardless of malaria rapid diagnostic test or microscopy result. The malaria result reflects the TAC target result

CI: confidence interval, OR: odds ratio, TAC: TaqMan Array Card

### Positive and negative predictive values of clinical and surveillance malaria test results

Of the inpatients assessed for AFI, 4,916 (57.9%) had documentation of clinical malaria testing (3,097 by microscopy and 1,819 by RDT). Using TAC PCR as the reference standard (n = 5,303 inpatients), the positive predictive value (PPV) for both microscopy and RDTs was substantially higher for surveillance tests compared to routine clinical tests (Microscopy: 94.4% vs. 78.6%; RDT: 95.4% vs. 88.9%). While the negative predictive value (NPV) was equivalent for surveillance and clinical microscopy (68.0% for both), surveillance RDT had a higher NPV than clinical RDT (81.7% vs. 74.7%). Both PPV and NPV varied by epidemic zone (Table 4).

**Table 4:** Positive and negative predictive values of malaria microscopy and RDTs in surveillance and clinical setting using PCR as a gold standard among inpatients by malaria epidemic zone

Epidemic zone*	Total^†^	Microscopy		RDT	
		Surveillance (%)	Clinical (%)	Surveillance (%)	Clinical (%)
*Positive predictive value*^‡^					
Overall	5303	94.4	78.6	95.4	88.9
Lake endemic	1068	92.7	79.1	96.6	83.8
Coast endemic	715	96.2	80.5	92.5	**
Highland epidemic prone	245	95.8	69.6	91.5	**
Seasonal	1615	95.8	76.5	96.5	90.9
Low risk	1660	95.0	87.9	91.7	80.8
*Negative predictive value*^¶^					
Overall	5303	68.0	68.0	81.7	74.7
Lake endemic	1068	47.6	63.0	74.3	75.9
Coast endemic	715	78.7	66.0	79.3	**
Highland epidemic prone	245	79.6	78.6	83.2	**
Seasonal	1615	60.3	72.5	76.9	74.3
Low risk	1660	87.0	83.5	88.8	73.3

*Epidemic zones were defined as: *Lake endemic* (8): Bungoma, Busia, Homa Bay, Kakamega, Kisumu, Migori, Siaya, Vihiga; *Coast endemic (5)*: Kilifi, Kwale, Lamu, Mombasa, Taita Taveta; *Highland epidemic prone (10)*: Bomet, Elgeyo-Marakwet, Kericho, Kisii, Narok, Nandi, Nyamira, Trans Nzoia, Uasin Gishu, West Pokot; *Seasonal (14)*: Baringo, Embu, Garissa, Isiolo, Kajiado, Kitui, Mandera, Marsabit, Meru, Samburu, Tana River, Tharaka-Nithi, Turkana, Wajir; *Low risk (10)*: Kiambu, Kirinyaga, Laikipia, Machakos, Makueni, Murang’a, Nairobi, Nakuru, Nyandarua, Nyeri

^†^Total number of persons with acute febrile illness with a PCR test done by TaqMan Array Card

^‡^Positive predictive value was calculated as the number of positive malaria microscopy (or RDT) results also positive by PCR (i.e., true positives) divided by the total number of positive malaria microcopy (or RDT) results (true positives plus false positives)

^¶^Negative predictive value was calculated as the number of negative malaria microscopy (or RDT) results also negative by PCR (i.e., true negatives) divided by the total number of negative malaria microcopy (or RDT) results (true negatives plus false negatives)

**Censored because fewer than 5 RDTs were captured from these epidemic zones in these data

PCR: polymerase chain reaction; RDT: malaria rapid diagnostic test

### Adherence to Kenya malaria clinical guidelines for management of severe malaria

Among 4,916 inpatients who received clinical malaria testing (microscopy or RDT), 2,094 (44.6%) tested positive. Of these positive cases, 1,847 (91.7%) had documentation of receiving antimalarials (Table 5). However, only 285 (15.4%) received the complete national guideline regimen for severe malaria (IV artesunate followed by AL). The majority (63.0%) were documented to have only received artesunate monotherapy. Quinine, used alone or in combination with artesunate or AL, accounted for 19.0% of treatments and was primarily used in a facility in Kakuma, the site of a refugee camp, in the seasonal malaria epidemic zone (Table 6).

**Table 5:** Antimalarials documented as used among those with positive malaria test

Antimalarial(s)	**N = 1847**
Artesunate therapy	
Monotherapy	1164 (63.0%)
+Artemether-lumefantrine (AL)	285 (15.4%)
+Quinine	15 (0.8%)
+AL and Quinine	15 (0.8%)
Quinine therapy	
Monotherapy	128 (6.9%)
+AL	193 (10.4%)
AL monotherapy	46 (2.5%)
Other	1 (0.1%)

**Table 6:** Antimalarial usage by epidemic zone

Epidemic zone^†^	Artesunate N = 1479	AL* N = 539	Quinine* N = 351
Lake endemic	669 (45.2%)	68 (12.6%)	1 (0.3%)
Coast endemic	86 (5.8%)	24 (4.5%)	0 (0.0%)
Highland epidemic prone	109 (7.4%)	2 (0.4%)	2 (0.6%)
Seasonal	499 (33.7%)	394 (73.1%)	348 (99.1%)
Low risk	116 (7.8%)	51 (9.5%)	0 (0.0%)

*Result not available for three patients

^†^Epidemic zones were defined as: *Lake endemic* (8): Bungoma, Busia, Homa Bay, Kakamega, Kisumu, Migori, Siaya, Vihiga; *Coast endemic (5)*: Kilifi, Kwale, Lamu, Mombasa, Taita Taveta; *Highland epidemic prone (10)*: Bomet, Elgeyo-Marakwet, Kericho, Kisii, Narok, Nandi, Nyamira, Trans Nzoia, Uasin Gishu, West Pokot; *Seasonal (14)*: Baringo, Embu, Garissa, Isiolo, Kajiado, Kitui, Mandera, Marsabit, Meru, Samburu, Tana River, Tharaka-Nithi, Turkana, Wajir; *Low risk (10)*: Kiambu, Kirinyaga, Laikipia, Machakos, Makueni, Murang’a, Nairobi, Nakuru, Nyandarua, Nyeri

Multiple discharge diagnoses were possible, but a malaria diagnosis was included on the discharge records for 90.1% of inpatients with a positive clinical malaria test, while the remaining 9.9% received a non-malaria diagnosis (e.g., pneumonia, meningitis, gastroenteritis). Notably, diagnostic inconsistency was observed: 15.0% of patients discharged with a malaria diagnosis had a negative malaria test, and 6.6% had no documented clinical malaria test at all.

While most patients who tested positive for malaria by clinical test received antimalarials (91.7%), antimalarial treatment was also administered to patients with a negative malaria test (17.2%) or no documented test (7.5%) (Table 7). Regarding antibiotics, approximately half of patients with a positive clinical malaria test also received them (55.8%), with higher proportions in the high transmission Lake (61%) and Coast (60%) endemic zones. Antibiotic use was generally high across all testing groups, with 89.3% of negative patients and 93.8% of untested patients receiving treatment.

**Table 7:** Receipt of antimalarials, antibiotics and discharge diagnosis by clinical malaria test result

	Tested for malaria as part of clinical care			Not tested for malaria
	Positive N = 2,094	Negative N = 2,598	Result not recorded N = 224	N = 3,569
Antimalarial medications given (n miss: 324)				
No	168 (8.3%)	2,039 (82.8%)	96 (46.6%)	3,216 (92.5%)
Yes	1,849 (91.7%)	423 (17.2%)	110 (53.4%)	260 (7.5%)
Antibiotic medications given (n miss: 324)				
No	892 (44.2%)	264 (10.7%)	58 (28.2%)	215 (6.2%)
Yes	1,125 (55.8%)	2,198 (89.3%)	148 (71.8%)	3,261 (93.8%)
Discharge diagnosis				
Malaria*	1,887 (90.1%)	390 (15.0%)	115 (51.3%)	235 (6.6%)
Other diagnosis	207 (9.9%)	2,208 (85.0%)	109 (48.7%)	3,334 (93.4%)

*Could include additional diagnoses such as pneumonia, meningitis, etc.

Among patients with a positive clinical malaria test, treatment with antimalarial medications was associated with lower odds of severe illness and mortality, although the 95% CI just crosses 1.0 (Table 8). Furthermore, patients who received a non-malaria diagnosis had increased odds of both severe illness and mortality compared to those diagnosed with malaria only. While patients with malaria plus another discharge diagnosis had greater odds of severe illness than those with malaria alone, this association did not extend to mortality.

**Table 8:** Disease severity and outcomes by antimalarial use and discharge diagnosis among patients with acute febrile illness and a positive malaria test during clinical care

	Not severe, N = 1506	Severe, N = 588	OR (95% CI)	Discharged, N = 2012	Dead, N = 15	OR (95% CI)
Antimalarials given during hospitalization (n miss = 77)						
No	105 (7%)	63 (11%)	Ref	127 (7%)	3 (20%)	Ref
Yes	1340 (93%)	509 (89%)	0.63 (0.46, 0.88)	1811 (93%)	12 (80%)	0.28 (0.08, 1.01)
Discharge diagnosis						
Malaria diagnosis only^†^	1021 (68%)	220 (37%)	Ref	1228 (61%)	5 (33%)	Ref
Malaria plus another diagnosis	365 (24%)	281 (48%)	3.57 (2.89, 4.43)	625 (31%)	5 (33%)	1.96 (0.57, 6.81)
Other diagnosis	93 (6%)	73 (12%)	3.64 (2.59, 5.11)	157 (8%)	4 (27%)	6.26 (1.66, 23.55)
Unknown diagnosis	27 (2%)	14 (2%)	2.41 (1.21, 4.59)	2 (0%)	1 (7%)	112.80 (9.53, 1582.32)

*Outcome missing for 67 patients

CI: Confidence interval, OR: odds ratio, Ref: referent level

## Discussion

Using the rich clinical data from the IFBS platform in Kenya, this study characterized the profile of hospitalized malaria patients, investigated factors associated with severe disease and mortality, and assessed adherence to MoH clinical management guidelines.

Consistent with established knowledge, [22, 23] the youngest age group (≤ 1 year) was associated with increased odds of developing severe illness. Higher odds of severe illness and mortality were also observed among patients with malnutrition and HIV-positive status, though the 95% CI crossed 1.0, likely due to small sample sizes. This is consistent with the prevailing assumption that a weakened immune system resulted in worse clinical outcomes [24–26].

Contrary to an expected protective effect, self-reported antimalarial treatment received in the week prior to presentation appeared as a risk factor severe illness or mortality. This paradoxical association may stem from inappropriate dosing, substandard drug quality, poor drug absorption, drug resistance or delays in care-seeking. Similarly, a history of seeking prior care was associated with severe illness and death (though the 95% CI crossed 1.0, likely due to small sample size). In Kenya, approximately 40–50% of patients seek initial care for malaria in the private sector [27], especially in unregulated drug dispensaries, where adherence to the national malaria treatment guidelines is not guaranteed and patients may receive substandard care—such as ineffective antimalarial therapy or an incomplete treatment course due to cost limitations.

Most cases treated in an outpatient setting will likely experience clinical resolution, thus never requiring hospitalization and subsequent presentation to a higher-level facility and become part of the IFBS database, resulting in a selection bias where the self-report to have received care at another facility or taken antimalarials prior to admission appeared as a potential risk factor associated with severe illness or mortality.

Delay in care-seeking, evidenced by longer fever duration and referral from a lower-level facility, was significantly associated with increased odds of severe illness and mortality [28–30].These findings underscore the importance of behavior change strategies and community messaging to promote timely care-seeking, appropriate referrals, and follow-up, especially for the youngest children. Messaging should emphasize completing a full 3-day course of oral artemisinin-based combination therapy (ACT) for uncomplicated malaria, ideally sourced from a regulated facility. Furthermore, while malaria diagnosis is routine for providers in endemic areas, diagnostic delays are a risk in non-endemic settings, potentially leading to severe disease, particularly since residents in these areas may lack immunity to malaria.

The TAC results for parasitologically confirmed malaria cases identified *non-typhoidal Salmonella* is the most common co-infection with malaria. At 2%, this rate was lower than what has been extensively documented in the literature [31, 32]. Other co-infecting pathogens of interest included various zoonotic agents such as *Rickettsia*, *Brucella*, *Bartonella*, *Leptospira*, *Coxiella burnetii,* and Rift Valley Fever. While any one of these could potentially result in severe illness [33], the data of this study showed that co-infection with malaria was not definitively associated with greater severe illness or mortality. (Odds ratio for mortality for malaria co-infections was 2.0, 95% CI crossing 1.0). A crucial limitation is that a positive PCR result alone does not confirm the true cause of AFI, as it may represent low-level or asymptomatic parasitaemia, complicating the interpretation of co-infection findings. When expanding the analysis to all patients with AFI (not limited to malaria), severe illness and mortality odds were higher among patients who had a non-falciparum infection, as well as among those with a negative TAC result.

### Accuracy of malaria diagnostic testing and adherence to national treatment guidelines

This project provided a unique opportunity to conduct an internal validation of clinical diagnostic methods by comparing facility-level microscopy and RDT results against PCR. Microscopy at the facility level suggests a substantial rate of false positivity—approximately one in five smears called positive when PCR was negative. This finding is corroborated by the consistently higher PCR positive concordance observed with positive smears read by surveillance staff who are certified at expert level. Potential causes include suboptimal equipment, reagents, contamination of stains and/or slides with dust or debris, or insufficient routine training. The high smear false positivity rate (~ 20%) in the lake endemic zone is particularly concerning, as this area accounts for over 80% of Kenya’s malaria burden and is the target of significant investments in ensuring quality malaria diagnostics and case management. Additionally, the lowest NPV of microscopy was observed in this same zone, likely reflecting the high prevalence of sub-microscopic parasitemia detected by PCR.

The findings of this study suggest malaria is over-diagnosed [34, 35], when relying solely on microscopy alone, which is the standard at many higher-level facilities. This underscores the critical need to strengthen quality-assured microscopy programmes through adequate microscopist training, properly functioning equipment/reagents, and robust EQA with routinely feedback. While over-diagnosis (false positives) may be preferred over under-diagnosis, it leads to wasted resources, unnecessary antimalarial exposure, and the risk of masking co-existing, non-malarial diagnoses that require different therapies.

The findings regarding adherence to the national malaria treatment guidelines in Kenya are both encouraging and revealing, offering valuable insights to inform malaria programming improvements. A key advantage of this study was the unique opportunity to directly assess adherence to the national treatment guidelines for patients hospitalized with malaria, supplementing the data collected through routine annual Health Facility Assessments (HFAs) conducted by the Kenya National Malaria Control Programme (NMCP).

While the IFBS data cannot be directly compared to the HFA results due to methodological differences, they highlight critical gaps in adherence to treatment guidelines. First, clinical malaria testing was documented in only 58% of AFI patients (compared to 83% in the 2023 HFA). However, the proportion of test-positive patients receiving antimalarials was high (92% in IFBS vs. 94% in HFA severe cases). Conversely, 17% of test-negative patients received treatment in IFBS, compared to 46% in the HFA cohort with severe malaria.

Critically, despite the national guidelines mandating IV artesunate followed by a course of AL for severe malaria, only 15.4% of antimalarial-treated patients were documented to have received the full regimen. The majority received artesunate monotherapy, a practice that directly contravenes standard guidelines and risks promoting artemisinin resistance [36].This is particularly concerning given the global emergence of artemisinin resistance, comproming the efficacy of first-line therapy, and underscoring the high drug pressure resulting from monotherapy [37, 38]. Adherence to AL completion was low across endemic zones, peaking at only 47.0% in the seasonal epidemic zone and dropping to 9.9% in the Lake endemic zone. Although the analysis is limited by documentation completeness—as patients may have been discharged with a prescription to take a 3-day course of AL—the documented low completion rate underscores a potential critical adherence gap that may benefit from enhanced training to ensure successful parasite clearance.

The significant use of IV quinine instead of first-line IV artesunate in the seasonal epidemic zone—primarily observed at the Kakuma refugee facility—suggests commodity stock challenges. Monitoring health facility readiness to stock life-saving treatments like IV artesunate is crucial, especially in non-endemic or low-immunity areas where patients are at a higher risk of developing severe disease.

In line with WHO recommendations for severe malaria in high to moderate transmission settings [20], antibiotics were widely used (82%) among patients admitted with AFI. Patients who had a positive malaria test were less likely to receive antibiotics than those who had a negative test or no malaria test. This makes sense from a clinical decision-making standpoint, where patients with a diagnosis of malaria were less likely to receive empiric antibiotics compared to those lacking a clear diagnosis. Unsurprisingly, antibiotics were also more frequently initiated for patients who developed severe illness or died, presumably due to clinical deterioration.

This study was subject to several limitations. First, using data abstracted from patient records, our criteria for severe malaria relied on clinical signs and symptoms suggestive of severe respiratory or neurologic involvement, as well as severe anaemia based on haemoglobin level of < 7 g/dL. Other laboratory values typically used to diagnose severe malaria, such as lactate levels and chemistry panels were typically unavailable. As such, this approach may have resulted in an underestimation of severe illness. Furthermore, the mortality analysis was limited by a small number of deaths, making statistically meaningful conclusions about risk factors difficult. This could have result from recruitment lag, as patients who died within the first hours of presentation were often missed. Statistically, clustering by surveillance site was not accounted for due to the low counts of some clusters. Finally, our analysis assumes that the abstracted data (e.g., antimalarial medication) accurately reflects the care received, meaning that our findings are susceptible to incomplete medical record documentation or data extraction errors.

The findings identify several key programmatic challenges across the malaria care continuum. Care-seeking delays are contributing to severe illness and mortality [39], underscoring the need to encourage timely presentation at formal healthcare settings. In diagnosis, there is likely significant over-diagnosis of malaria by microscopy, necessitating strengthened training and EQA programme.

The AFI cases that are potentially misdiagnosed as malaria may result in higher mortality if appropriate therapies, such as failure to initiate broad-spectrum antibiotics, are withheld, since the data show that antibiotic initiation rates are lower among those with diagnosis of malaria. This is supported by our co-infection data, where the detection of a non-*Plasmodium* pathogen (such as NTS) was associated with higher mortality odds. Failure to provide empiric antibiotics for severe malaria due to reliance on a false positive microscopy result may lead to the missed treatment of a bacterial aetiology, thereby increasing the risk of death.

Once diagnosed, there are also concerning gaps: only a small fraction of patients completed the required full course of antimalarials (IV artesunate followed by AL), and the persistent use of IV quinine highlights a need to ensure consistent stock availability of IV artesunate, particularly in seasonal epidemic zones.

This analysis highlights the value of an integrated disease surveillance platform, even when implemented at a limited number of facilities, in identifying malaria-specific programmatic challenges. To enhance representativeness and long-term sustainability, routine inpatient data collected through electronic medical records are needed to capture malaria case information to support both clinical and programmatic decision-making.

## Supplementary Information

Additional file1 (DOCX 19 kb)

## Data Availability

Data cannot be shared publicly because they are bound by Government of Kenya provisions, including the Data Protection Act of 2019. Data are available from KEMRI via the Data Governance Committee (contact via email [cghr@kemri.go.ke] (mailto:cghr@kemri.go.ke) or telephone + 254-20-22923) to researchers who meet the criteria for access to confidential data and with permission of Kenya Ministry of Health.

## References

[1] Kenya Malaria Indicator Survey, 2010. https://dhsprogram.com/pubs/pdf/mis7/mis7.pdf. Accessed 22 July 2025.

[2] Kenya Malaria Indicator Survey, 2020. https://dhsprogram.com/pubs/pdf/MIS36/MIS36.pdf. Accessed 22 July 2025.

[3] Gething PW, Casey DC, Weiss DJ, Bisanzio D, Bhatt S, Cameron E, et al. Mapping *Plasmodium falciparum* mortality in Africa between 1990 and 2015. N Engl J Med. 2016;375:2435–45. 27723434 10.1056/NEJMoa1606701 PMC5484406

[4] Njuguna P, Maitland K, Nyaguara A, Mwanga D, Mogeni P, Mturi N, et al. Observational study: 27 years of severe malaria surveillance in Kilifi, Kenya. BMC Med. 2019;17:124. 31280724 10.1186/s12916-019-1359-9 PMC6613255

[5] Kapesa A, Kweka EJ, Atieli H, Afrane YA, Kamugisha E, Lee MC, et al. The current malaria morbidity and mortality in different transmission settings in Western Kenya. PLoS ONE. 2018;13:e0202031. 30092043 10.1371/journal.pone.0202031 PMC6084967

[6] Akech S, Chepkirui M, Ogero M, Agweyu A, Irimu G, English M, et al. The clinical profile of severe pediatric malaria in an area targeted for routine RTS,S/AS01 malaria vaccination in Western Kenya. Clin Infect Dis. 2020;71:372–80. 31504308 10.1093/cid/ciz844 PMC7353324

[7] Githinji S, Oyando R, Malinga J, Ejersa W, Soti D, Rono J, et al. Completeness of malaria indicator data reporting via the District Health Information Software 2 in Kenya, 2011–2015. Malar J. 2017;16:344. 28818071 10.1186/s12936-017-1973-y PMC5561621

[8] Ayieko P, Ogero M, Makone B, Julius T, Mbevi G, Nyachiro W, et al. Characteristics of admissions and variations in the use of basic investigations, treatments and outcomes in Kenyan hospitals within a new clinical information network. Arch Dis Child. 2016;101:223–9. 26662925 10.1136/archdischild-2015-309269 PMC4789757

[9] Mbondji PE, Kebede D, Soumbey-Alley EW, Zielinski C, Kouvividila W, Lusamba-Dikassa PS. Health information systems in Africa: descriptive analysis of data sources, information products and health statistics. J R Soc Med. 2014;107:34–45. 24914127 10.1177/0141076814531750 PMC4109358

[10] Kniss JM, Kibaba G, Baguma E, Bhattarai Chhetri S, Hendren C, Ntaro M, et al. Quality of care and post-discharge morbidity among children diagnosed with severe malaria in rural Uganda: a prospective cohort study. PLoS Glob Public Health. 2024;4:e0003794. 39374246 10.1371/journal.pgph.0003794 PMC11458001

[11] Kenya Malaria Programme Review 2018, Republic of Kenya, Ministry of Health. ihttps://measuremalaria.cpc.unc.edu/wp-content/uploads/2019/11/tr-19-3191.pdf

[12] Verani JR, Eno EN, Hunsperger EA, Munyua P, Osoro E, Marwanga D, et al. Acute febrile illness in Kenya: clinical characteristics and pathogens detected among patients hospitalized with fever, 2017–2019. PLoS ONE. 2024;19:e0305700. 39088453 10.1371/journal.pone.0305700 PMC11293630

[13] Church J, Maitland K. Invasive bacterial co-infection in African children with *Plasmodium falciparum* malaria: a systematic review. BMC Med. 2014;12:31. 24548672 10.1186/1741-7015-12-31 PMC3928319

[14] Phu NH, Day NPJ, Tuan PQ, Mai NTH, Chau TTH, Van Chuong L, et al. Concomitant bacteremia in adults with severe falciparum malaria. Clin Infect Dis. 2020;71:e465–70. 32107527 10.1093/cid/ciaa191 PMC7713686

[15] Scott JA, Berkley JA, Mwangi I, Ochola L, Uyoga S, Macharia A, et al. Relation between falciparum malaria and bacteraemia in Kenyan children: a population-based, case-control study and a longitudinal study. Lancet. 2011;378:1316–23. 21903251 10.1016/S0140-6736(11)60888-X PMC3192903

[16] WHO. Malaria microscopy quality assurance manual: version 2. Geneva, World Health Organization, 2016. https://www.who.int/docs/default-source/documents/publications/gmp/malaria-microscopy-quality-assurance-manual.pdf. Accessed 23 July 2025.

[17] Zurovac D, Rowe AK. Quality of treatment for febrile illness among children at outpatient facilities in sub-Saharan Africa. Ann Trop Med Parasitol. 2006;100:283–96. 16762109 10.1179/136485906X105633

[18] WHO. Giemsa Staining of Malaria Blood Films: SOP. . Geneva, World Health Organization, 2016. https://www.who.int/docs/default-source/wpro---documents/toolkit/malaria-sop/gmp-sop-07a.pdf. Accessed 23 July 2025.

[19] Guidelines for the Diagnosis, Treatment and Prevention of Malaria in Kenya, Division of National Malaria Programme, Ministry of Health, Kenya, 6th Edition, Revised 2020

[20] CDC: Treatment of Severe Malaria https://www.cdc.gov/malaria/hcp/clinical-guidance/treatment-of-severe-malaria.html. Accessed 23 July 2025.

[21] [21WHO. Guidelines for malaria. Geneva, World Health Organization, 2024. https://www.who.int/publications. Accessed 23 July 2025.

[22] Garcia KKS, Laporta GZ, Duarte EC, Soremekun S, Abrahão AA, da Silva AC, et al. Towards malaria elimination: a nationwide case-control study to assess risk factors for severe malaria-related deaths in Brazil. Trop Med Int Health. 2025;30:1194–210. 40990168 10.1111/tmi.70028 PMC12588806

[23] Reyburn H, Mbatia R, Drakeley C, Bruce J, Carneiro I, Olomi R, et al. Association of transmission intensity and age with clinical manifestations and case fatality of severe *Plasmodium falciparum* malaria. JAMA. 2005;293:1461–70. 15784869 10.1001/jama.293.12.1461

[24] Figueroa-Romero A, Saura-Lázaro A, Fernández-Luis S, González R. Uncovering HIV and malaria interactions: the latest evidence and knowledge gaps. Lancet HIV. 2024;11:e255–67. 38458223 10.1016/S2352-3018(24)00035-3

[25] Mahittikorn A, Kotepui KU, De Jesus Milanez G, Masangkay FR, Kotepui M. A meta-analysis on the prevalence and characteristics of severe malaria in patients with *Plasmodium* spp. and HIV co-infection. Sci Rep. 2021;11:16655. 34404814 10.1038/s41598-021-95591-6 PMC8371128

[26] Berkley JA, Bejon P, Mwangi T, Gwer S, Maitland K, Williams TN, et al. HIV infection, malnutrition, and invasive bacterial infection among children with severe malaria. Clin Infect Dis. 2009;49:336–43. 19548833 10.1086/600299 PMC2853703

[27] Odhiambo FO, O’Meara WP, Abade A, Owiny M, Odhiambo F, Oyugi EO. Adherence to national malaria treatment guidelines in private drug outlets: a cross-sectional survey in the malaria-endemic Kisumu County, Kenya. Malar J. 2023;22:307. 37821868 10.1186/s12936-023-04744-7 PMC10568760

[28] Mpimbaza A, Ndeezi G, Katahoire A, Rosenthal PJ, Karamagi C. Demographic, socioeconomic, and geographic factors leading to severe malaria and delayed care seeking in Ugandan children: a case-control study. Am J Trop Med Hyg. 2017;97:1513–23. 29016322 10.4269/ajtmh.17-0056 PMC5817743

[29] Agimas MC, Aweke MN, Mengistu B, Baffa LD, Fentie EA, Shewarega ES, et al. Prevalence and associated factors of delay in seeking malaria treatment among under five children in the Horn of Africa: a systematic review and meta-analysis. PLoS ONE. 2025;20:e0333593. 41004556 10.1371/journal.pone.0333593 PMC12468861

[30] da Silva AC, Duarte EC, Marchesini PB, Viana GMR, Ramalho WM, Garcia KKS. Factors associated with hospitalizations due to severe malaria in the non-endemic Brazilian region: a case-control study in the extra-Amazon Region from 2011 to 2019. Malar J. 2025;24:227. 40652249 10.1186/s12936-025-05357-y PMC12255989

[31] Wilairatana P, Mala W, Klangbud WK, Kotepui KU, Rattaprasert P, Kotepui M. Prevalence, probability, and outcomes of typhoidal/non-typhoidal *Salmonella* and malaria co-infection among febrile patients: a systematic review and meta-analysis. Sci Rep. 2021;11:21889. 34750425 10.1038/s41598-021-00611-0 PMC8576030

[32] Hogan B, Eibach D, Krumkamp R, Sarpong N, Dekker D, Kreuels B, et al. Malaria coinfections in febrile pediatric inpatients: a hospital-based study from Ghana. Clin Infect Dis. 2018;66:1838–45. 29408951 10.1093/cid/cix1120 PMC5982794

[33] Mandage R, Kaur C, Pramanik A, Kumar V, Kodan P, Singh A, et al. Association of dengue virus and *Leptospira* co-infections with malaria severity. Emerg Infect Dis. 2020;26:1645–53. 32687019 10.3201/eid2608.191214 PMC7392441

[34] Harchut K, Standley C, Dobson A, Klaassen B, Rambaud-Althaus C, Althaus F, et al. Over-diagnosis of malaria by microscopy in the Kilombero Valley, Southern Tanzania: an evaluation of the utility and cost-effectiveness of rapid diagnostic tests. Malar J. 2013;12:159. 23663437 10.1186/1475-2875-12-159 PMC3689637

[35] Kahama-Maro J, D’Acremont V, Mtasiwa D, Genton B, Lengeler C. Low quality of routine microscopy for malaria at different levels of the health system in Dar es Salaam. Malar J. 2011;10:332. 22047131 10.1186/1475-2875-10-332 PMC3217957

[36] WHO. Artemisinin resistance and artemisinin-based combination therapy efficacy: Status report. Geneva, World Health Organization, 2018. https://www.who.int/docs/default-source/documents/publications/gmp/who-cds-gmp-2018-26-eng.pdf. Accessed 24 July 2025.

[37] Rosenthal PJ, Asua V, Bailey JA, Conrad MD, Ishengoma DS, Kamya MR, et al. The emergence of artemisinin partial resistance in Africa: how do we respond? Lancet Infect Dis. 2024;24:e591–600. 38552654 10.1016/S1473-3099(24)00141-5 PMC12954456

[38] WHO. Strategy to respond to antimalarial drug resistance in Africa. Geneva, World Health Organization, 2022. https://www.who.int/publications/i/item/9789240060265. Accessed 20 Aug 2025.

[39] Mousa A, Al-Taiar A, Anstey NM, Badaut C, Barber BE, Bassat Q, et al. The impact of delayed treatment of uncomplicated *P. falciparum* malaria on progression to severe malaria: a systematic review and a pooled multicentre individual-patient meta-analysis. PLoS Med. 2020;17:e1003359. 33075101 10.1371/journal.pmed.1003359 PMC7571702

