# Supplementary material for: Using sentinel surveillance system data to characterize severe malaria illness and quality of malaria case management among hospitalized patients in Kenya, 2017–2024
Source: Malar J. 2026 Jan 14;25:90. doi: 10.1186/s12936-025-05738-3 (PMC12888659; doi:10.1186/s12936-025-05738-3)
Supplement: Supplementary file 1 — Additional file1 (DOCX 19 kb) [file 12936_2025_5738_MOESM1_ESM.docx]

# **Supplemental table 1. Sample size of acute febrile illness patients by surveillance site**

| Surveillance site | Start year | End year | All patients with AFI, n | | | | Patients with positive RDT ± microscopy, n (%) | | |
| --- | --- | --- | --- | --- | --- | --- | --- | --- | --- |
|  |  |  | Total | RDT | Microscopy | PCR | Total | RDT | Microscopy |
| Busia CRH | 2021 | NA | 1131 | 1131 | 1123 | 455 | 509 (0.45) | 509 (0.45) | 505 (0.45) |
| Coast General TRH (Mombasa) | 2018 | NA | 905 | 892 | 587 | 725 | 200 (0.22) | 200 (0.22) | 120 (0.20) |
| JJaramogi Oginga Odinga TRH (Kisumu) | 2022 | NA | 409 | 400 | 388 | 193 | 163 (0.40) | 163 (0.41) | 157 (0.40) |
| Kakamega CRH | 2017 | 2021 | 562 | 530 | 448 | 429 | 274 (0.49) | 272 (0.51) | 227 (0.51) |
| Kakuma MH | 2017 | 2021 | 2099 | 1972 | 1267 | 1301 | 759 (0.36) | 757 (0.38) | 503 (0.40) |
| Kapenguria CRH | 2021 | NA | 374 | 370 | 366 | 236 | 109 (0.29) | 108 (0.29) | 107 (0.29) |
| Kenyatta National Hospital (Nairobi) | 2017 | 2023 | 756 | 653 | 12 | 646 | 42 (0.06) | 41 (0.06) | 2 (0.17) |
| Loitokitok SCH | 2021 | NA | 443 | 442 | 422 | 244 | 6 (0.01) | 6 (0.01) | 6 (0.01) |
| Makueni CRH | 2021 | NA | 567 | 565 | 194 | 358 | 21 (0.04) | 21 (0.04) | 19 (0.10) |
| Mama Lucy Hospital (Nairobi) | 2021 | NA | 689 | 685 | 637 | 193 | 60 (0.09) | 60 (0.09) | 57 (0.09) |
| Marsabit CRH | 2020 | NA | 926 | 917 | 917 | 750 | 25 (0.03) | 25 (0.03) | 24 (0.03) |
| Nakuru CRH | 2021 | NA | 575 | 570 | 567 | 539 | 64 (0.11) | 64 (0.11) | 62 (0.11) |
| CRH: County referral hospital; PCR: polymerase chain reaction; RDT: malaria rapid diagnostic test; TRH: teaching and referral hospital | | | | | | | | | |

## **Supplemental table 2. TaqMan Array Card multiplex PCR test targets by time period**

| June 2017 to early November 2020 | November 2020 to July 2024 |
| --- | --- |
| *Bartonella* spp.  *Brucella* spp.  *Coxiella burnetii*  Crimean-Congo haemorrhagic fever virus  Chikungunya virus  Dengue virus  Ebola virus  Ebola (Bundibugyo) virus  Ebola (Sudan) virus  Hepatitis E virus  Lassa virus  *Leishmania* spp.  *Leptospira* spp.  Marburg virus  Nipah virus  O'nyong'nyong virus  Plasmodium  *Rickettsia* spp.  Rift Valley Fever virus  *Salmonella* spp.  *Salmonella*Typhi  HIV I  HIV II  *Trypanosoma brucei*  West Nile virus  *Yersinia pestis*  Yellow fever virus  Zika virus | Targets from 2017-2020 plus:  *Burkholderia pseudomallei*  *Orientia tsutsugamushi*  Oropouche virus  *Plasmodium falciparum*  *Plasmodium vivax*  *Streptococcus pneumoniae*  *Salmonella* Paratyphi A |

## **Supplemental Table 3. Component variables for severe illness composite variable by age group**

|  | Age group (years), n (%) | | | |
| --- | --- | --- | --- | --- |
| Variable | ≤1, N = 188 | 1-4, N = 1,134 | 5-19, N = 681 | ≥20, N = 194 |
| Severe illness | 73 (38.8%) | 390 (34.4%) | 225 (33.0%) | 25 (12.9%) |
| Hypoxaemia (n miss: 1)* | 20 (10.7%) | 99 (8.7%) | 38 (5.6%) | 5 (2.6%) |
| Stridor (n miss: 3) | 7 (3.7%) | 17 (1.5%) | 4 (0.6%) | 0 (0.0%) |
| Nasal flaring (n miss: 934)^†^ | 5 (2.7%) | 17 (1.6%) | NA | NA |
| Lower chest indrawing (n miss: 898)^†^ | 8 (4.3%) | 15 (1.3%) | NA | NA |
| Grunting (n miss: 934)^†^ | 5 (2.7%) | 11 (1.0%) | NA | NA |
| Consciousness (n miss: 1)^‡^ | 1 (0.5%) | 43 (3.8%) | 36 (5.3%) | 6 (3.1%) |
| Convulsions (n miss: 1) | 8 (4.3%) | 45 (4.0%) | 23 (3.4%) | 2 (1.0%) |
| Nuchal rigidity (n miss: 3) | 1 (0.5%) | 7 (0.6%) | 8 (1.2%) | 1 (0.5%) |
| Photophobia (n miss: 4) | 1 (0.5%) | 2 (0.2%) | 1 (0.1%) | 0 (0.0%) |
| Bulging fontanelle (n miss: 2,016)^¶^ | 1 (0.6%) | NA | NA | NA |
| Petechial rash (n miss: 18) | 2 (1.1%) | 5 (0.4%) | 2 (0.3%) | 0 (0.0%) |
| Jaundice (n miss: 1) | 4 (2.1%) | 56 (4.9%) | 78 (11.5%) | 10 (5.2%) |
| Severe anaemia (n miss: 929)** | 40 (34.2%) | 209 (29.6%) | 121 (32.0%) | 7 (10.4%) |
| *Defined as oxygen saturation ≤90% or a supplemental oxygen requirement ^†^Only asked of children aged ≤ 5 years ^‡^Defined as a level of consciousness below “alert” on the Alert/Verbal/Pain/Unresponsive [AVPU] scale ^¶^Only asked of children age ≤ 1 year **Defined as haemoglobin value <7g/dL NA: not applicable | | | | |
